# Editorial: The Role of Complement in Tumors

**DOI:** 10.3389/fimmu.2020.00139

**Published:** 2020-02-11

**Authors:** Barbara E. Rolfe, Ruben Pio, Trent M. Woodruff, Maciej M. Markiewski, Helga D. Manthey

**Affiliations:** ^1^Australian Institute for Bioengineering and Nanotechnology, The University of Queensland, Brisbane, QLD, Australia; ^2^Center for Applied Medical Research (CIMA), University of Navarra, Pamplona, Spain; ^3^School of Biomedical Sciences, The University of Queensland, Brisbane, QLD, Australia; ^4^Department of Immunotherapeutics and Biotechnology, School of Pharmacy, Texas Tech University Health Sciences Center, Abilene, TX, United States

**Keywords:** complement, cancer, metastasis, C5b-9, C1q, C3a, C5a, complement regulatory proteins

Activation of the complement system is one of the earliest responses to invading pathogens and tissue damage (1). Complement activation leads to production of a range of effectors including the opsonin C3b, the anaphylatoxins C3a and C5a, and the C5b-9 complex (membrane attack complex; MAC) (2, 3). In addition to potent innate immune activities, complement effector systems also contribute to efficient adaptive immune responses (4). While critical to proper immune function, inappropriate or excessive complement activation contributes to many pathological inflammatory conditions (5), including cancer. As described in this issue, the complement system is increasingly recognized as a double-edged sword: on the one hand contributing to the anti-tumor response, but on the other protecting the tumor against immune attack and promoting metastasis.

## Complement-Dependent Cytotoxicity and C5b-9 (Membrane Attack Complex)

As described by Macor et al., the complement system has long been recognized to contribute to anti-tumor defense mechanisms via complement dependent cytotoxicity (CDC) (6) and antibody-dependent cell mediated cytotoxicity (ADCC) (7). The introduction of recombinant antibodies for cancer treatment has led to renewed interest in complement as an anti-tumor defense system. The protective role of complement in cancer is discussed, with focus on the beneficial effect of complement-fixing antibodies which initiate cancer cell killing via CDC.

The cytotoxic activities of C5b-9, and the mechanisms by which it damages cancer cells, are further discussed by Fishelson and Kirschfink, along with the multiple mechanisms that tumor cells employ to resist C5b-9-induced death. They discuss the potential for therapeutic approaches to counter tumor escape mechanisms and potentiate antibody-based immunotherapies, but caution that intervention strategies to augment complement activation could also worsen outcomes.

Although C5b-9 has traditionally been attributed an anti-tumoral role through CDC, Vlaicu et al. review the evidence that C5b-9 at a sub-lytic dose stimulates tumor growth. Hence strategies to counteract the tumor-promoting traits of C5b-9 and potentiate anti-tumoral actions (including enhanced efficacy of antibody-based immunotherapy) may be the next major direction for immuno-oncology.

## Complement Regulatory Proteins

As described by Geller and Yan, membrane and soluble complement regulatory proteins (CRPs) prevent excessive complement activation. Therefore, over-expression of CRPs by tumor cells protects them against complement-mediated attack, interferes with anti-tumor therapies, and enhances metastatic potential. The application of CRPs as prognostic biomarkers and therapeutic targets is discussed, along with the potential for combinatorial approaches with other anti-tumor therapies.

## Complement C1q

The first subcomponent of the classical complement pathway, C1q is a pattern recognition molecule locally synthesized by macrophages and dendritic cells (8). Bioinformatics analysis by Mangogna et al. suggests C1q as a new prognostic biomarker for several cancers.

## Anaphylatoxins C3a AND C5a

Since the seminal paper of Markiewski et al. (9) identifying a role for C5a in promoting tumor progression, similar effects have been demonstrated in a range of murine cancer models. Wang et al. propose C3aR and C5aR1 as a new class of immune checkpoints. They discuss findings suggesting that C3aR/C5aR signaling regulates T cell mediated antitumor immunity via transcriptional suppression of interleukin (IL)-10. Given resistance of the majority of patients to the current forms of immunotherapy, and adverse reactions associated with these approaches, the authors suggest the manipulation of the C3aR/C5aR/IL-10 pathway as an alternative strategy for cancer.

Lenkiewicz et al. discovered that C3 and C5 cleavage fragments enhance trafficking, motility and, therefore, dissemination of malignant cells in hematologic malignancies through a p38 MAPK and inducible heme oxygenase 1 (HO-1) manner. They propose that activation of the complement cascade in patients with these malignancies (e.g., triggered by infection) can contribute to faster dissemination of disease. Thus, targeting this pathway may ameliorate dissemination of leukemic cells and improve clinical outcomes in these patients.

Kleczko et al. discuss the potential for complement targeting therapies for the treatment of intractable cancers, in particular lung cancer. They review the mechanisms by which the anaphylatoxins C3a and C5a influence tumor growth and promote epithelial-mesenchymal transition and tumor metastasis. Since complement proteins can regulate both pro- and anti-tumorigenic pathways, the authors stress the need to better understand the effects of complement activation within tumor tissue, and how this may be influenced by different oncogenic drivers.

Cancer metastasis is estimated to be responsible for greater than 90% of cancer deaths (10). As discussed by Ajona et al., distorted complement homeostasis not only remodels the tumor microenvironment by inhibiting anti-tumor immune responses, but is also crucial to metastasis, endowing tumor cells with properties required for metastatic dissemination and establishment. Complement activation products (primarily C3a and C5a) induce a range of mediators which promote epithelial-mesenchymal transition, tumor growth, invasion, dissemination via lymphatic and circulatory systems, and also protect cells within the metastatic niche. The authors highlight the potential of complement-targeting drugs to augment the clinical efficacy of current immunotherapies and effectively eradicate both primary tumors and distant metastases.

## Conclusions

Despite recent clinical advances in cancer immunotherapy, the estimated percentage of patients responding to checkpoint inhibitors in the United States in 2018 was only 12.46% (11). Hence there remains an urgent need for novel therapeutic strategies to boost response rates. As a critical link between the innate and adaptive immune systems, the complement system is a promising therapeutic target. However, knowledge is the key to realizing the clinical potential of complement-targeting therapeutics. Only a thorough understanding of the role of complement pathways in the tumor microenvironment will enable development of strategies to selectively (and safely) target the pro-tumor effects of complement, while simultaneously augmenting the anti-tumor effects. To quote Fishelson and Kirschfink, “currently we perceive only the tip of the ice-berg of … interactions between cancer cells and complement components.” Indeed, given the diversity of responses, therapeutic protocols will likely need to be optimized for each cancer type and stage, and possibly for individual cancer patients, depending on their “immune history.”

## Author Contributions

All authors contributed to the conception and design of this work. BR drafted the manuscript, the other editors RP, TW, MM, and HM provided critical revisions and approved the submitted manuscript.

### Conflict of Interest

The authors declare that the research was conducted in the absence of any commercial or financial relationships that could be construed as a potential conflict of interest.
